# Prognostic value of patient‐derived xenograft engraftment in pediatric sarcomas

**DOI:** 10.1002/cjp2.210

**Published:** 2021-04-09

**Authors:** Helena Castillo‐Ecija, Guillem Pascual‐Pasto, Sara Perez‐Jaume, Claudia Resa‐Pares, Monica Vila‐Ubach, Carles Monterrubio, Ana Jimenez‐Cabaco, Merce Baulenas‐Farres, Oscar Muñoz‐Aznar, Noelia Salvador, Maria Cuadrado‐Vilanova, Nagore G Olaciregui, Leire Balaguer‐Lluna, Victor Burgueño, Francisco J Vicario, Alejandro Manzanares, Alicia Castañeda, Vicente Santa‐Maria, Ofelia Cruz, Veronica Celis, Andres Morales La Madrid, Moira Garraus, Maite Gorostegui, Margarita Vancells, Rosalia Carrasco, Lucas Krauel, Ferran Torner, Mariona Suñol, Cinzia Lavarino, Jaume Mora, Angel M Carcaboso

**Affiliations:** ^1^ Institut de Recerca Sant Joan de Deu Barcelona Spain; ^2^ Department of Pediatric Oncology Hospital Sant Joan de Deu Barcelona Spain; ^3^ Department of Pediatric Surgery Hospital Sant Joan de Deu Barcelona Spain; ^4^ Department of Pediatric Orthopedic Surgery Hospital Sant Joan de Deu Barcelona Spain; ^5^ Department of Pathology Hospital Sant Joan de Deu Barcelona Spain

**Keywords:** patient‐derived xenograft, Ewing sarcoma, rhabdomyosarcoma, osteosarcoma, prognosis

## Abstract

The goals of this work were to identify factors favoring patient‐derived xenograft (PDX) engraftment and study the association between PDX engraftment and prognosis in pediatric patients with Ewing sarcoma, osteosarcoma, and rhabdomyosarcoma. We used immunodeficient mice to establish 30 subcutaneous PDX from patient tumor biopsies, with a successful engraftment rate of 44%. Age greater than 12 years and relapsed disease were patient factors associated with higher engraftment rate. Tumor type and biopsy location did not associate with engraftment. PDX models retained histology markers and most chromosomal aberrations of patient samples during successive passages in mice. Model treatment with irinotecan resulted in significant activity in 20 of the PDXs and replicated the response of rhabdomyosarcoma patients. Successive generations of PDXs responded similarly to irinotecan, demonstrating functional stability of these models. Importantly, out of 68 tumor samples from 51 patients with a median follow‐up of 21.2 months, PDX engraftment from newly diagnosed patients was a prognostic factor significantly associated with poor outcome (*p* = 0.040). This association was not significant for relapsed patients. In the subgroup of patients with newly diagnosed Ewing sarcoma classified as standard risk, we found higher risk of relapse or refractory disease associated with those samples that produced stable PDX models (*p* = 0.0357). Overall, our study shows that PDX engraftment predicts worse outcome in newly diagnosed pediatric sarcoma patients.

## Introduction

Pediatric sarcomas are a rare and heterogeneous group of skeletal and soft tissue malignancies accounting for approximately 12% of all childhood solid tumors [1]. The most frequently occurring are Ewing sarcoma, osteosarcoma, and rhabdomyosarcoma, altogether representing 13% of all malignant tumors in patients younger than 14 years, and up to 18% of all tumors among patients between 15 and 19 years [2, 3, 4]. With up‐to‐date treatment modalities, the 5‐year survival rates have improved in the last decade to 79% for Ewing sarcoma, 73% for osteosarcoma, and 70% for rhabdomyosarcoma [5, 6, 7]. Unfortunately, intensive and multimodal treatments currently used in pediatric sarcomas have topped their efficacy [1, 8, 9]. A substantial proportion of patients relapse and do not respond consistently to rescue chemotherapies because of tumor evolution into a chemoresistant phenotype [10].

Patient‐derived xenograft (PDX) tumor models in immunodeficient mice are well‐recognized tools for the study of rare tumors and their treatment [11, 12, 13]. In colorectal cancer, epithelial ovarian cancer, and lung adenocarcinoma, for instance, PDXs retain the main properties of the original patient tumor, including histology, genetic and genomic alterations, gene expression profile, and heterogeneity [6, 11, 14, 15]. Successful engraftment of a patient tumor biopsy in animals might predict the outcome of patients [16]. For breast, pancreatic, and lung cancers, engraftment in mice is associated with worse patient prognosis [15, 17, 18]. Likely, there is a selection process enabling the most aggressive clones to expand and prevail in successive passages in mice [11, 13, 19, 20, 21]. PDX models of most pediatric sarcomas are suitable for such establishment [22, 23, 24]. Whether the parameter ‘PDX engraftment’ could be useful for identifying pediatric sarcoma patients at risk of relapse or refractory disease is not well characterized. To address this question, here we establish PDX models from Ewing sarcoma, osteosarcoma, and rhabdomyosarcoma patients and include available clinical data along with the PDX data [25]. To characterize this new preclinical platform, we address whether PDX tumors retained the main histologic, genomic, and functional properties of patient tumors during successive passages in mice. Sample characterization includes histopathology markers, chromosomal profiles (copy number alteration [CNA]), and preclinical treatment assays (efficacy of irinotecan).

## Materials and methods

### Xenotransplantation of patient samples

The appropriate Institutional Review Board approved the research protocol associated with this project (M‐1608‐C). Patients or their legal guardians signed informed consent at Sant Joan de Déu Hospital (SJD, Barcelona, Spain). Clinical information parameters obtained from patients are listed in supplementary material, Table S1. Patients were biopsied using either a surgical procedure (open surgery or minimally invasive surgery) or percutaneous tissue core biopsy with a 18G Tru‐Cut needle (Argon Medical Devices, Athens, TX, USA). Biopsies were used fresh or were cryopreserved. For the cryopreservation process, tissues were minced as 2 × 2 × 1 mm pieces with a scalpel on sterile petri dishes. Three to four of these pieces were added to 2 ml cryogenic vials (Corning, Glendale, AZ, USA) containing 1.5 ml of inactivated fetal bovine serum with 10% dimethyl sulfoxide (Life Technologies, Grand Island, NY, USA). Vials were stored overnight in a freezing container (Cool Cell; Corning) at −80 °C and then transferred to liquid nitrogen for long‐term storage.

Work with mice adhered to the European regulations and was approved by the animal experimental ethics committee at the Universitat de Barcelona (animal protocol numbers 135/11 and 134/18). In brief, we implanted patient tumor biopsies (freshly excised or cryopreserved) subcutaneously in 4–6‐week‐old female NOD‐SCID mice (Envigo, Barcelona, Spain) as previously described [26]. Tumors engrafting successfully were named F0 generation. When tumor volume achieved 1,000–1,500 mm^3^, we performed passage of freshly excised tissue to athymic nude mice (Envigo). We used NOD‐SCID mice for the passage of specific rhabdomyosarcoma models. We named subsequent generations with the number of the passage (e.g. F1 was the first filial generation after initial F0 engraftment). Information parameters obtained from successfully engrafted PDX models are listed in supplementary material, Table S2.

To interrogate whether clinical parameters of the patient disease, including the sample collection method (surgical biopsy versus Tru‐Cut needle), correlated with the likelihood of successful PDX engraftment, we used mixed‐effects logistic regression analysis and calculated the odds ratios and their corresponding 95% confidence intervals (CIs). To dichotomize age, we computed an optimum threshold by maximizing the Youden index using negative or positive engraftment [27].

### Analysis of fusion genes

We amplified Ewing sarcoma fusion gene types *EWSR1*‐*ERG* and *EWSR1*‐*FLI1*, and rhabdomyosarcoma fusion genes *PAX3*‐*FKHR* and *PAX7*‐*FKHR* with the polymerase chain reaction (PCR). The sequences of the primers were *EWSR1* forward, 5′‐TCCTACAGCCAAGCTCCAAGTC‐3′; *FLI1* reverse, 5′‐GTGTCAGGCATGGAGGATGGA‐3′; *ERG* reverse, 5′‐GAGAAGGCATATGGCTGGTGG‐3′; *PAX3* forward, 5′‐AGGCATGGATTTTCCAGCTATA‐3′; *PAX7* forward, 5′‐TCTGCCTACGGAGCCCG‐3′; and *FKHR* reverse, 5′‐GGGACAGATTATGACGAATTGAATT‐3′. Ewing sarcoma fusion genes were detected in agarose gels, while rhabdomyosarcoma fusion genes were quantified with a real‐time quantitative PCR technique, using the probe FAM‐5′‐CCGGTCAGCAACGGCCTGTCT‐3′.

### Histopathology

During the process of PDX establishment, the original patient tumors and PDX models at different passages must be characterized and compared [16]. First, we compared histology on hematoxylin and eosin staining, and the expression of tumor‐related proteins in the patient tumor biopsies and PDX tumors. To analyze whether protein expression (detected by immunostaining) changed upon successive mouse‐to‐mouse transplantation, we studied PDX tissues at three different filial generations: F0, as the first generation in mice, and F2 and F5, as early and late successive passages in mice. Tissues were fixed overnight in buffered formalin and embedded in paraffin. To stain human tumor cells in engrafted tumor tissues, we used anti‐human nuclei primary antibody (1:200, MAB4383; Merck Millipore, Burlington, MA, USA). To detect proliferating cells, we used anti‐Ki67 antibody (1:200, ACK02; Leica Biosystems, Wetzlar, Germany). We stained proteins highly expressed by specific tumor types, such as CD99 for Ewing sarcoma (1:20, NCL‐L‐CD99‐187; Leica Biosystems), MyoD1 for rhabdomyosarcoma (1:20, M3512; Agilent Technologies, Santa Clara, CA, USA), and the secreted protein acidic and rich in cysteine (SPARC) for osteosarcoma (1:80, 35‐5500; Invitrogen, Carlsbad, CA, USA).

### PDX growth

We measured tumor growth rate as the time needed to achieve the experimental endpoint, i.e. tumor volume of 1,500 mm^3^. Tumor volume was calculated as (length × width^2^)/2, length being the longitudinal diameter and width the transverse diameter of the subcutaneous tumor.

### Analysis of CNAs

We compared the CNA profiles of tumor biopsies and PDX tissue at generations F2 and F5. We performed whole‐genome analysis using the high‐density array CytoScan® HD platform (Affymetrix, Thermo Fisher Scientific, Waltham, MA, USA) as previously described [10]. Total DNA from frozen samples was digested, ligated, PCR‐amplified and purified, fragmented, biotin‐labeled, and hybridized according to manufacturer's instructions. We analyzed the CytoScan® HD Array data with the Chromosome Analysis Suite (ChAS) software (Affymetrix, Thermo Fisher Scientific).

### Irinotecan activity *in vivo*

We used 20 of the new PDX models to evaluate the activity of irinotecan, as a model drug with established activity against pediatric solid tumors including sarcomas [10, 28, 29]. We reasoned that if the PDXs were truly representative of the patients' diseases, they would likely respond to this drug and such response should remain stable over successive PDX generations. Among the patients from which these PDX models were established, 11 Ewing sarcoma PDXs resulted from eight patients who received irinotecan as part of their rescue treatment (combined with temozolomide, with or without additional vincristine or trabectedin); 7 rhabdomyosarcoma PDXs from six patients who received irinotecan as part of the upfront treatment of the primary tumor (either alone or combined with carboplatin or vincristine); and 2 osteosarcoma PDXs from two patients who did not receive irinotecan. To compare responses to treatment of patients and PDXs, we selected newly diagnosed patients treated with irinotecan as upfront treatment and with available PDX models from their primary tumor. We evaluated treatment response in such patients using the responsive evaluation criteria in solid tumors (RECIST) protocol or the metabolic tumor volume.

For all efficacy studies in mice, we inserted freshly excised PDX tumors (obtained from one mouse of the immediate earlier generation) in both flanks of athymic nude or NOD‐SCID mice. Upon engraftment (tumor volumes ranging 100–500 mm^3^), mice were distributed to control or treatment groups, with care that tumor volume means and standard deviations (STDEV) in both groups were similar. Treatment groups received one cycle of 10 mg/kg/day irinotecan (Hospira, Lake City, IL, USA) in a 5‐day‐on‐2‐off regimen, intraperitoneal, for two consecutive weeks, as previously described [30]. Control groups received saline using the same regimen as treated groups. Tumor volume was measured three times a week, until day 14, in which response to treatment was evaluated as previously described [10]. We defined complete response (CR) as tumor mass <50 mm^3^ and >50% reduction at the end of treatment (day 14); partial response (PR) as tumor volume regression ≥50% at day 14 but tumor volume ≥50 mm^3^; stable disease (SD) as <50% regression and ≤25% increase in initial volume at day 14; and progressive disease as <50% regression from initial volume and >25% increase in initial volume at day 14.

To address whether the response to the drug changed upon consecutive passages of the PDX in mice, we evaluated the response to irinotecan of different filial generations in three of the models, one early after initial engraftment (*F* ≤ 2) and the second one later (*F* ≥ 6). After one cycle of irinotecan, we followed up all animals weekly until tumor regrowth to endpoint (1,500 mm^3^) or day 100 to build Kaplan–Meier curves and estimate median survival times. We used the log‐rank test to compare survival curves between treatments and between different generations from the same PDX model.

### Prognostic value of positive tumor engraftment

The main purpose of this work was to address whether successful engraftment in mice of tumor biopsies of newly diagnosed or relapsed Ewing sarcoma, rhabdomyosarcoma, and osteosarcoma patients had a prognostic effect on overall survival (OS) and event‐free survival (EFS) of these patients.

To assess the prognostic effect of successful PDX engraftment on EFS and OS of the biopsied children, we used Cox models with a robust sandwich variance estimator to account for the correlation of the data. We calculated hazard ratios (HR) and their corresponding 95% CI. We determined median EFS and OS with the Kaplan–Meier method. For each patient, we calculated EFS as the time from biopsy until date of patient recurrence, death, or last follow‐up, and OS as the time from biopsy until patient's death or last follow‐up.

To address whether PDX engraftment of primary tumors predicted prognosis of patients classified as ‘standard risk’ according to clinical methods, Ewing sarcoma patients whose biopsies were obtained at initial diagnosis (diagnostic cohort) were divided into two groups, standard risk and high risk, according to their risk stratification following the clinical trial GEIS21 [5]. For each of such patient groups, we studied the association between the variables PDX engraftment and relapse, using the Fisher's exact test.

### Statistics

We performed statistical analyses using GraphPad Prism 8 software (Graphpad, La Jolla, CA, USA) and R software version 3.5.2 (R Foundation for Statistical Computing, Vienna, Austria).

## Results

### Patients

We included 51 children, 37 males, and 14 females in this study, with newly diagnosed or relapsed Ewing sarcoma (*N* = 31), osteosarcoma (*N* = 10), or rhabdomyosarcoma (*N* = 10, five each embryonal and alveolar), from November 2010 to November 2019. Median age at inclusion was 11.4 years (range: 0.52–17.9). Most patients (73%) were nonmetastatic at diagnosis. Details on tumor types, fusion genes, anatomic location of primary tumors, risk stratification, and presence of metastases are in supplementary material, Tables S3 (Ewing sarcoma), S4 (osteosarcoma), and S5 (rhabdomyosarcoma).

### Biopsies and PDXs

We obtained 68 biopsy samples. Engraftment outcomes of individual patient samples are in supplementary material, Table S6. Forty‐one tumor samples were Ewing sarcoma, 12 osteosarcoma, and 15 rhabdomyosarcoma. In total, 44% of samples engrafted efficiently in mice, with engraftment rates for each tumor type shown in Table 1. Age older than 12 years and relapse were factors associated with increased engraftment rate when pooling all diseases together (Table 1). Tumor type, biopsy location, and presence of metastases at diagnosis were not associated with engraftment rate. Procedural factors such as sample conservation (freshly excised or cryopreserved) and application of Matrigel (Corning, Glendale, AZ, USA) to the sample inserted in the mice did not affect engraftment rate. Biopsies obtained by surgery or by the Tru‐Cut method engrafted with a similar success rate (see supplementary material, Table S7).

**Table 1:** Association of patient factors with engraftment.

Factor	No. of Samples	Engrafted (%)	No. of Engrafted	Odds Ratio (95% CI)	*P*
Age (years)					
<12	36	30.6	11		
≥12	32	59.4	19	3.88 (1.1–14.3)	0.042
Tumor type					
All	68	44.1	30		
Ewing sarcoma	41	41.5	17		
Osteosarcoma	12	41.7	5	0.99 (0.2–5.6)^*^	
Rhabdomyosarcoma	15	53.3	8	1.97 (0.4–10.7)^*^	0.69
Alveolar	9	66.7	6		
Embryonal	6	33.3	2		
Timing of surgery					
Diagnosis	31	22.6	7		
Relapse	37	62.2	23	15.4 (1.3–182.8)	0.031
Biopsy origin					
Limbs	22	31.8	7		
Head and neck	14	64.3	9	21.1 (0.04–11 056.5)^†^	
Chest wall and ribs	7	42.9	3	3.4 (0.05–215.9)^†^	
Lung or pleura	13	61.5	8	15 (0.07–3,075.8)^†^	
Pelvic bones	3	33.3	1	1.2 (0.01–134.4)^†^	0.17
Muscle	2	100	2	‡	
Testes	1	0	0	‡	
Vertebral spine	6	0	0	‡	
Metastasis at diagnosis					
No	48	37.5	18		
Yes	20	60.0	12	3.62 (0.64–20.50)	0.15

* Compared to Ewing sarcoma.

† Compared to limbs.

‡ Excluded from statistical analysis of biopsy origin due to lack of an event in the number of engrafted or nonengrafted samples.

Of the 51 patients, 15 had two or more biopsies included in this study. Nine of them produced two stable PDXs. We studied whether the engraftment of a first biopsy from one patient predicted engraftment of successive biopsies of the same patient at more advanced stages of the disease. We found that 9 out of 11 such second biopsies engrafted after a first biopsy with positive PDX engraftment, but the association between first and second engraftments was not statistically significant, likely due to the low number of cases in the study (see supplementary material, Table S8).

Because Ewing sarcoma patients were predominant in the study cohort, we also analyzed their data separately. We found that age older than 12 years was not determinant in this separate analysis, while relapse and the presence of metastases at diagnosis were significantly associated with engraftment (see supplementary material, Table S9).

### Comparative histopathology of patient tumors and PDX samples

**Figure 1:**
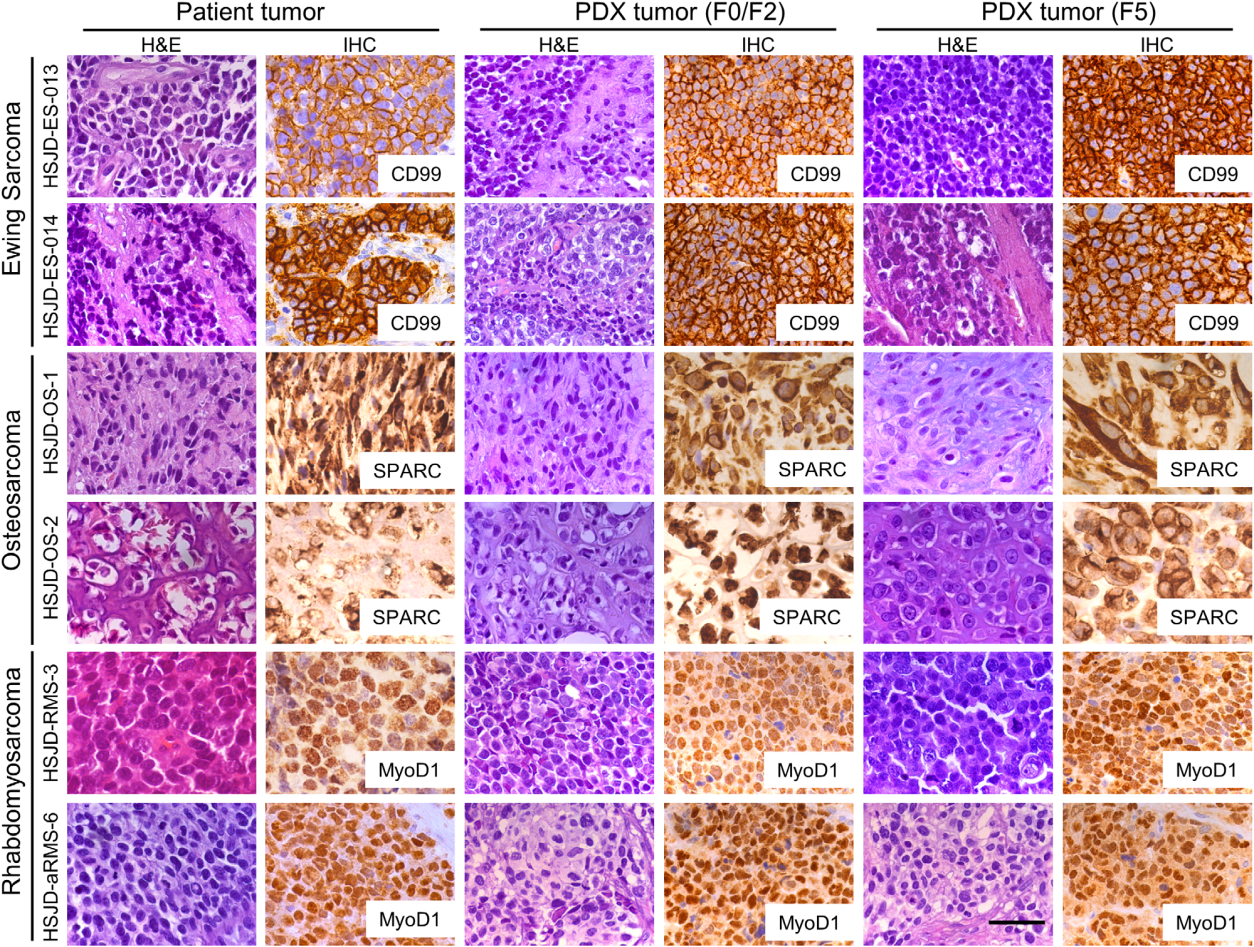
Comparative histology (hematoxylin and eosin and IHC staining) of six representative cases of original human tumor biopsies and the corresponding PDXs at early passages (F0/F2) and late passage (F5). CD99 (cell membrane), SPARC (cytoplasm), and MyoD1 (nuclear) are stained in brown. These representative samples were selected from six Ewing sarcomas, three osteosarcomas, and three rhabdomyosarcomas with complete histopathology studies. All images were obtained using a microscope at ×40 objective magnification. Scale bar represents 50 μm.

Histopathology of the tumors and expression of tumor markers CD99, SPARC, and MyoD1 did not change significantly upon successive mouse‐to‐mouse passaging in 12 PDX models studied by IHC (Figure 1). The typical small‐blue‐round‐cell tumor and histologic architecture of the original biopsies remained preserved in Ewing sarcoma xenografts at early and late passages. Stroma around Ewing sarcoma and rhabdomyosarcoma tumor cells, and osteoid in osteosarcoma tumors, did not change significantly upon engraftment. Anti‐human nuclear antigen staining was positive in all samples (see supplementary material, Figure S1). Tumor anatomy and histology markers were similar in PDX pairs established from the same patients (see supplementary material, Figure S2).

### Tumor growth rate

The number of proliferating cells (% of Ki67‐positive nuclei by immunohistochemistry (IHC); representative images in supplementary material, Figure S3A and quantification in supplementary material, Figure S3B) in patient biopsies was 54 ± 26% (mean and STDEV of *N* = 12 cases). Their corresponding PDX samples at passage F0 showed a lower number of stained cells (37 ± 19%). PDX tumors at generations higher than F0 showed increasing number of Ki67‐positive cells (40 ± 23% at F2 and 44 ± 21% at F5; see supplementary material, Figure S3B). Although the differences in Ki67 staining were not statistically significant, tumors at F5 achieved endpoint significantly faster (53 ± 21 days; mean ± STDEV of *N* = 20 cases) than at the earlier stage F2 (92 ± 47 days; see supplementary material, Figure S3C).

### CNA in patient biopsies and corresponding PDXs

We selected one Ewing sarcoma tumor with low CNAs and one rhabdomyosarcoma with highly aberrant chromosomic profile for CNA analyses. Karyotype alterations of Ewing sarcoma (HSJD‐ES‐012) were equivalent for patient tumor and PDX models at F2 and F5 [10]. Ewing sarcoma CNA included a copy‐neutral loss of heterogeneity in chromosomes 3p and 20q as previously reported [10] (see supplementary material, Figure S4). For rhabdomyosarcoma (HSJD‐RMS‐4), both PDX models, F2 and F5, shared 90% of the alterations observed in the patient tumor. Chromosomal aberrations shared by the biopsy and the PDX included whole gains in one or two copies of chromosomes 2, 3, 4, 6, 10, 11, 12, 14, 16, 17, 18, 19, 20, 21, and X; partial gain in chromosomes 1, 7, 9, and 13; and whole chromosome loss in chromosome 15. Nonequivalent alterations among biopsy and PDX included gain of two copies of chromosomes 5, 8, and 22 in the tumor biopsy, and partial gains of chromosomes 5 and 22 and one copy gain of chromosome 8 in both PDX models (see supplementary material, Figure S4).

### Antitumor activity of irinotecan in subcutaneous PDXs

**Figure 2:**
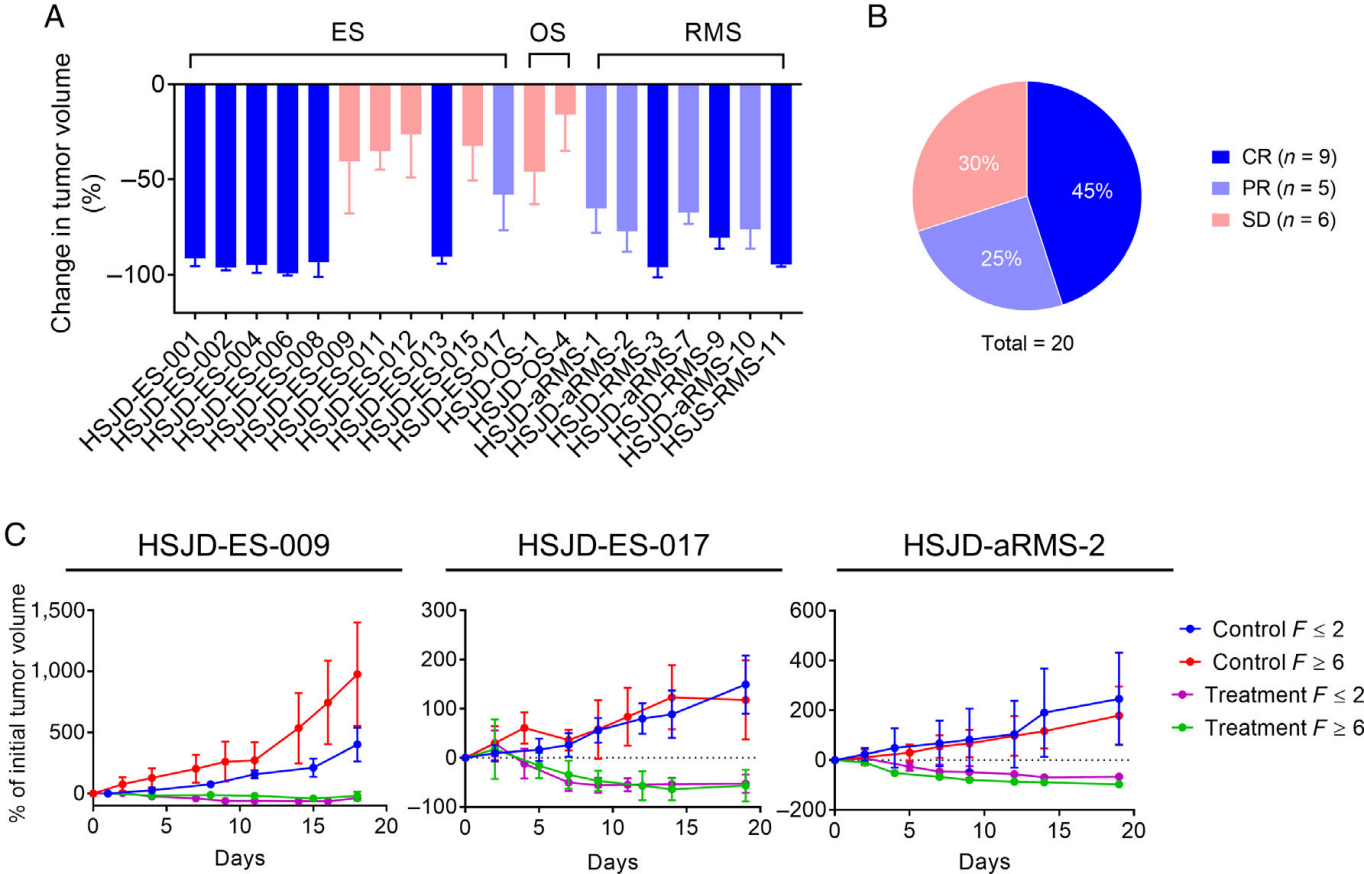
Antitumor activity of irinotecan in subcutaneous PDX. (A) Change in tumor volume (mean and STDEV of 3–15 tumors) at the end of irinotecan treatment (day 14). (B) Percentage of tumor models achieving each response. (C) Tumor volume (% of volume at treatment start) in three PDX pairs at passage *F* ≤ 2 or *F* ≥ 6, treated with one cycle of irinotecan (treatment) or not treated (control). Models and *F* were HSJD‐ES‐009 (F2 versus F6), HSJD‐ES‐017 (F1 versus F8) and HSJD‐aRMS‐2 (F2 versus F10).

All PDX models (see identification and clinical details in supplementary material, Table S10) had a measurable response after one cycle of irinotecan (Figure 2A and supplementary material, Table S11). We found that 9 of 20 models (45%) achieved CR to treatment and all achieved at least SD (Figure 2B).

We compared responses to treatment of patient and PDX of four newly diagnosed rhabdomyosarcoma patients receiving upfront irinotecan, three combined with carboplatin, and one with vincristine (see supplementary material, Table S12). The three patients treated with irinotecan plus carboplatin achieved the same response to treatment as their corresponding PDX models (HSJD‐aRMS‐7, PR; HSJD‐aRMS‐10, PR; and HSJD‐RMS‐11, CR) to irinotecan. The patient treated with irinotecan plus vincristine achieved a lower degree of response (SD) compared to the corresponding PDX (HSJD‐RMS‐9, CR).

In the PDX models for which irinotecan activity was evaluated at two different filial generations, both tumors at passages *F* ≥ 6 and *F* ≤ 2 showed similar responses to irinotecan (Figure 2C). Upon treatment cessation, the PDXs grew similarly, even those that achieved a CR, and achieved similar median survivals until endpoint (see supplementary material, Table S13).

### Prognostic value of positive PDX engraftment in mice

**Figure 3:**
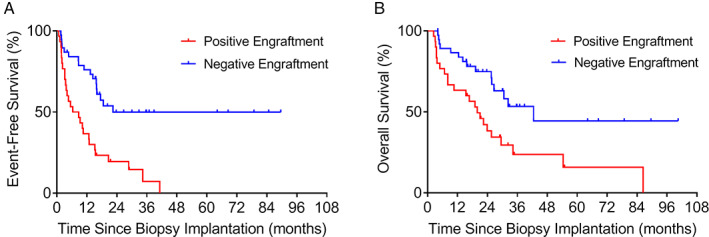
Kaplan–Meier estimation of (A) EFS and (B) OS among all patients with positive (*n* = 30) or negative (*n* = 38) engraftment of their PDX.

Patients included in the analysis had a median follow‐up of 21.2 months (range: 2.40–101 months). Figure 3 shows the outcome of the study cohort according to engraftment in mice. Median EFS for patients with positive engraftment (7.56 months) was significantly shorter than that of patients with negative engraftment (22.4 months; *p* = 0.00021; HR: 3.34; 95% CI: 1.77–6.31; Figure 3A). Similarly, median OS was shorter for patients with positive engraftment (19.9 months) compared to that of patients with negative engraftment (42.5 months; *p* = 0.0069; HR: 2.51; 95% CI: 1.29–4.89; Figure 3B).

**Figure 4:**
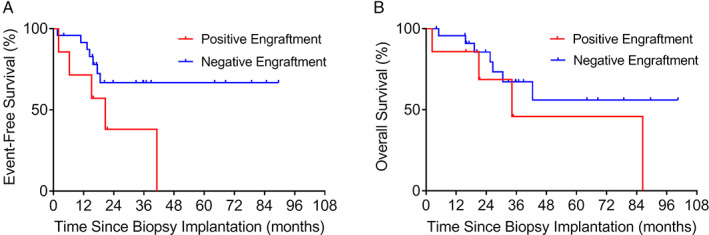
Kaplan–Meier estimation of (A) EFS and (B) OS among patients of the diagnostic cohort with positive (*n* = 7) or negative (*n* = 24) engraftment of their PDX.

Because relapse was associated with increased engraftment rate, we stratified patients into those whose samples were obtained at initial diagnosis (diagnostic cohort) and at relapse (relapse cohort). We further separated each cohort according to positive and negative engraftment. In the diagnostic cohort, positive engraftment associated with shorter median EFS (20.6 months). In contrast, median EFS was not reached for negative engraftment (*p* = 0.040; HR: 2.87; 95% CI: 1.05–7.85; Figure 4A). In this cohort, patients with positive engraftment also had shorter median OS (34.2 months), although not statistically significant compared to patients with negative engraftment (*p* = 0.19; HR: 2.06; 95% CI: 0.70–6.0; Figure 4B).

**Figure 5:**
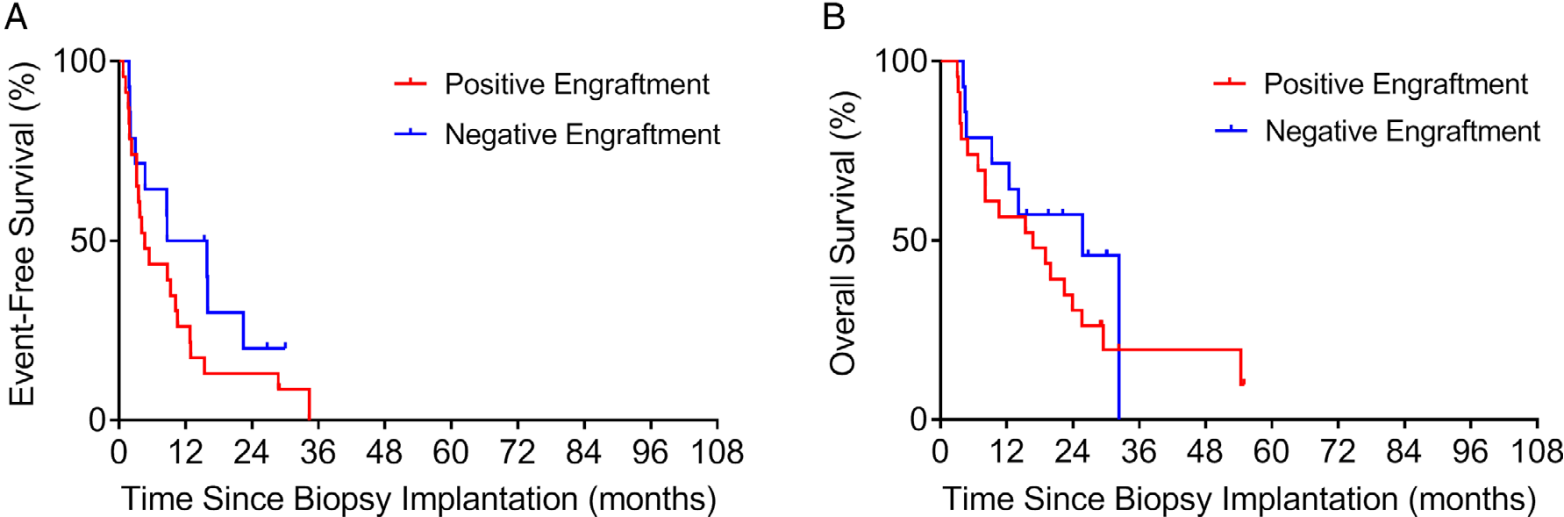
Kaplan–Meier estimation of (A) EFS and (B) OS among patients of the relapse cohort with positive (*n* = 23) or negative (*n* = 14) engraftment of their PDX.

In the relapse cohort, we did not find a significant association of positive and negative engraftment with median EFS of 4.56 and 12.2 months, respectively (*p* = 0.17; HR: 1.70; 95% CI: 0.80–3.63; Figure 5A) and OS of 16.7 and 25.7 months, respectively (*p* = 0.30; HR: 1.51; 95% CI: 0.69–3.32; Figure 5B).

We studied 18 newly diagnosed Ewing sarcoma patients, 7 of which were standard risk and 11 high risk, with follow‐up times of 15.5–101 months. Two out of the seven patients classified as standard risk relapsed and eventually died of disease. Biopsies from these two patients successfully established PDXs in mice, while biopsies from the remaining five standard risk patients did not. In fact, we found that positive PDX engraftment predicted relapse of patients classified as standard risk (*p* = 0.0357; Fisher's exact test). Among the 11 newly diagnosed Ewing sarcoma patients classified as high risk, two established PDX models. Both patients relapsed or were refractory to treatment and eventually died of disease. Among the remaining nine high‐risk patients, for whom PDX engraftment was unsuccessful, another two relapsed. The association of positive PDX engraftment and relapse was not significant in these high‐risk patients (*p* = 0.1091).

## Discussion

PDXs are powerful tools for cancer biology and precision cancer medicine studies, and may be useful to predict patient prognosis [17]. Determination of prognosis is especially relevant in pediatric patients, 80% of whom will survive cancer and reach adulthood [31]. Identification of prognostic factors for these children helps select appropriate treatment for their disease, ensuring maximum clinical benefit along with minimum long‐term toxicity [32]. In the current study, we show that positive engraftment of tumor biopsies in mice is a prognostic factor related to poorer EFS for newly diagnosed pediatric patients with the most frequent being bone or soft tissue sarcomas.

All patients in the diagnostic cohort whose samples engrafted in mice relapsed and died of their disease while we were performing this study. This discovery might be especially relevant for newly diagnosed Ewing sarcoma patients, because PDX engraftment predicted relapse in patients otherwise classified as standard risk at diagnosis. Thus, our results suggest that PDX engraftment might help unveil patients at higher risk and stratify them accordingly.

The engraftment rate calculated in this study, 44%, is similar to previous studies of pediatric solid malignancies both for primary and relapsed tumors [23, 33, 34]. Patient determinants favoring tumor biopsy engraftment in mice are not yet clear. We show here that age older than 12 years or relapsed disease correlates highly with pediatric sarcoma biopsy engraftment. Age and relapsed disease are also determining factors for engraftment of other tumors such as lung adenocarcinoma and hepatoblastoma [15, 35]. In adult patients, advanced disease stage increases the likelihood of PDX engraftment from patients with lung cancer [36]. Also in adult patients, tumor mutations, such as in *EGFR*, *BRCA1/BRCA2*, and *SMAD4* genes, decrease engraftment rates in adenocarcinoma, breast cancer, and pancreatic cancer, respectively [14, 15, 18].

Other studies have evaluated some of the technical factors addressed in our study, such as sample conservation. In agreement with our results, cryopreserved pancreatic cancer biopsies engrafted efficiently in mice [37]. We did not evaluate ‘engraftment site’ as a factor, but the orthotopic site is especially relevant to reproduce the complex microenvironment necessary for the growth of central nervous system tumors [38, 39, 40], and could facilitate and accelerate tumor implantation and recapitulate patient tumor characteristics [41, 42]. However, our selection of the subcutaneous site is justified by its advantages of offering a simple and fast surgical procedure and easier follow‐up of the mice, as it does not require imaging systems to visualize tumor site implantation [13].

Interestingly, we found that the Tru‐Cut technique, a less invasive method than the surgical collection of tumor samples, was successful for the purpose of PDX establishment, and resulted in more rapid patient recovery [43]. Patients for whom surgery would constitute an excessive risk or associated morbidity, such as relapsed patients with advanced disease, are more suitable for the Tru‐Cut method. On the other hand, the Tru‐Cut method is at a disadvantage, due to its resulting smaller sample size and tumor heterogeneity and necrosis in some tumor areas; therefore, the Tru‐Cut technique can result in greater challenges to pathologists and cancer biologists.

Our studies confirm the similarities of histology and genetics of the PDX and the original biopsy, consistent with previous data showing that CNA variations in patient biopsies were conserved in tumor xenografts of breast cancer and neuroblastoma [17, 44]. It was not within the scope of our project to perform a comprehensive comparison of paired PDX models coming from the same patient. In a previous study, we compared systematically Ewing sarcoma PDX pairs obtained from the same patients, finding functional differences among them that we attributed to patient treatments [10].

Other studies have shown changes in tumor architecture and chromosomal stability during successive passages over time that could differ from those arising in patients [13, 45]. Breast cancer tumors and their corresponding xenografts share mostly all single‐nucleotide variations, with the exception of some clusters that are dominant in the PDX but not in the tumor patients [17]. These results, among others, suggest that there exists clonal selection during xenograft establishment [21, 46, 47]. It is therefore not clear whether such alterations affect the capacity of the PDX to represent the original patient biopsy from the functional perspective, such as response to treatment. Our studies of irinotecan activity in PDX models at different stages of their engraftment partially addressed this question. We confirmed the stability of our models during successive engraftments and their ability to replicate patient response to treatment at the preclinical dosage of 10 mg/kg using the protracted dosing schedule [30]. It is likely that this dosage provides a higher SN‐38 systemic exposure than the one achievable in humans, due to the better conversion of the prodrug irinotecan in mice [48], which could explain the different response to irinotecan found between the PDX and one of our patients. Nevertheless, our data support that PDX might predict clinical efficacy of personalized medicine while keeping stable tumor architecture and heterogeneity [11, 12, 13]. Performing drug efficacy studies using the PDX models could aid in the design of personalized therapies for our patients. The main limiting factor is the lag time between the necessary time for the biopsy to engraft (9–17 weeks, according to our experience) and the time‐critical needs of the patients. Disease progression is usually faster than the time needed to engraft and expand tumors, and not all patients can benefit from this approach. According to our experience, we need at least 28 weeks to obtain preclinical results for candidate individualized treatments. This number is obtained by adding the median times to achieve engraftment of the F0 generation (13 weeks; range: 9–17 weeks), median times for expanding the F1 generation in a sufficient number of mice to start candidate treatments (13 weeks; range: 6–20 weeks), and times to treat and evaluate treatment activity (2 weeks typically). Although personalized treatments were not within the scope of our work, in our cohort approximately 50% (13) of patients with associated stable PDX progressed later than 28 weeks after biopsy, and could have obtained a theoretical benefit from preclinical assays using their PDX.

In conclusion, our study suggests that PDX engraftment at the time of patient diagnosis helps to identify aggressive pediatric sarcoma tumors with poorer prognosis, predicting disease progression. Such PDX models stably represent patient disease, both morphologically and functionally. Whether this systematic study of the PDXs from our patients has future clinical implications for personalized medicine, patient risk classification, and intensification of treatments should now be addressed in prospective clinical trials.

## Author contributions statement

All authors were involved in data collection and manuscript editing. HC‐E, SP‐J, CL and AMC analyzed the data. JM and AMC conceived the work, acquired funding, and provided resources. HC‐E and AMC wrote the original draft and generated the figures. AMC supervised the project.

## Supporting information


**Figure S1.** Staining of human cells in six representative cases of xenografts at early and late passages
**Figure S2.** Comparative histology of two PDX from the same rhabdomyosarcoma patient at relapse and necropsy
**Figure S3.** Analysis of cell proliferation in human tumor biopsies and their corresponding PDXs
**Figure S4**. Whole‐genome visualization of the chromosomal profiles of patients with Ewing sarcoma and rhabdomyosarcoma and their matched PDX models
**Table S1.** Clinical data of patients with Ewing sarcoma, osteosarcoma, and rhabdomyosarcoma
**Table S2.** Data from PDX established from patients with Ewing sarcoma, osteosarcoma, and rhabdomyosarcoma
**Table S3.** Clinical information from patients with Ewing sarcoma (*N* = 31; median age: 12.3 years; range: 0.52–17.9 years)
**Table S4.** Clinical information from patients with osteosarcoma (*N* = 10; median age: 11.1 years; range: 5.67–14.7 years)
**Table S5.** Clinical information from patients with rhabdomyosarcoma (*N* = 10; median age: 9.7 years; range: 4.03–15.4 years)
**Table S6.** Engraftment outcomes of patient samples
**Table S7.** Association of technical factors with engraftment
**Table S8.** Association of previous biopsy engraftment with the engraftment of the following biopsy obtained from the same patient
**Table S9.** Association of patient factors with engraftment in patients with Ewing sarcoma
**Table S10.** Clinical data from PDX models included in the efficacy study of irinotecan
**Table S11.** PDX response to treatment with a single cycle of irinotecan
**Table S12.** Comparative response to irinotecan‐based treatments of rhabdomyosarcoma patients and their corresponding PDX
**Table S13.** Comparative response to irinotecan treatment and median survival of PDXs at *F* ≤ 2 or *F* ≥ 6 passages

## References

[1] Pappo AS , Dirksen U . Rhabdomyosarcoma, Ewing sarcoma, and other round cell sarcomas. J Clin Oncol 2018; 36: 168–179. 29220292 10.1200/JCO.2017.74.7402

[2] Grünewald TGP , Cidre‐Aranaz F , Surdez D , *et al*. Ewing sarcoma. Nat Rev Dis Primers 2018; 4: 5. 29977059 10.1038/s41572-018-0003-x

[3] Kansara M , Teng MW , Smyth MJ , *et al*. Translational biology of osteosarcoma. Nat Rev Cancer 2014; 14: 722–735. 25319867 10.1038/nrc3838

[4] Skapek SX , Ferrari A , Gupta AA , *et al*. Rhabdomyosarcoma. Nat Rev Dis Primers 2019; 5: 1. 30617281 10.1038/s41572-018-0051-2 PMC7456566

[5] Mora J , Castañeda A , Perez‐Jaume S , *et al*. GEIS‐21: a multicentric phase II study of intensive chemotherapy including gemcitabine and docetaxel for the treatment of Ewing sarcoma of children and adults: a report from the Spanish sarcoma group (GEIS). Br J Cancer 2017; 117: 767–774. 28787430 10.1038/bjc.2017.252 PMC5589997

[6] de Alava E . Ewing sarcoma, an update on molecular pathology with therapeutic implications. Surg Pathol Clin 2017; 10: 575–585. 28797503 10.1016/j.path.2017.04.001

[7] Grohar PJ , Janeway KA , Mase LD , *et al*. Advances in the treatment of pediatric bone sarcomas. Am Soc Clin Oncol Educ Book 2017; 37: 725–735. 28561686 10.14694/EDBK_175378 PMC6066791

[8] Crompton BD , Stewart C , Taylor‐Weiner A , *et al*. The genomic landscape of pediatric Ewing sarcoma. Cancer Discov 2014; 4: 1326–1341. 25186949 10.1158/2159-8290.CD-13-1037

[9] Whelan JS , Davis LE . Osteosarcoma, chondrosarcoma, and chordoma. J Clin Oncol 2018; 36: 188–193. 29220289 10.1200/JCO.2017.75.1743

[10] Castillo‐Ecija H , Monterrubio C , Pascual‐Pasto G , *et al*. Treatment‐driven selection of chemoresistant Ewing sarcoma tumors with limited drug distribution. J Control Release 2020; 324: 440–449. 32497782 10.1016/j.jconrel.2020.05.032

[11] Hidalgo M , Amant F , Biankin AV , *et al*. Patient‐derived xenograft models: an emerging platform for translational cancer research. Cancer Discov 2014; 4: 998–1013. 25185190 10.1158/2159-8290.CD-14-0001 PMC4167608

[12] Gao H , Korn JM , Ferretti S , *et al*. High‐throughput screening using patient‐derived tumor xenografts to predict clinical trial drug response. Nat Med 2015; 21: 1318–1325. 26479923 10.1038/nm.3954

[13] Byrne AT , Alférez DG , Amant F , *et al*. Interrogating open issues in cancer precision medicine with patient‐derived xenografts. Nat Rev Cancer 2017; 17: 254–268. 28104906 10.1038/nrc.2016.140

[14] Eoh KJ , Chung YS , Lee SH , *et al*. Comparison of clinical features and outcomes in epithelial ovarian cancer according to tumorigenicity in patient‐derived xenograft models. Cancer Res Treat 2018; 50: 956–963. 29059719 10.4143/crt.2017.181 PMC6056987

[15] Stewart EL , Mascaux C , Pham NA , *et al*. Clinical utility of patient‐derived xenografts to determine biomarkers of prognosis and map resistance pathways in EGFR‐mutant lung adenocarcinoma. J Clin Oncol 2015; 33: 2472–2480. 26124487 10.1200/JCO.2014.60.1492

[16] Kresse SH , Meza‐Zepeda LA , Machado I , *et al*. Preclinical xenograft models of human sarcoma show nonrandom loss of aberrations. Cancer 2012; 118: 558–570. 21713766 10.1002/cncr.26276

[17] DeRose YS , Wang G , Lin YC , *et al*. Tumor grafts derived from women with breast cancer authentically reflect tumor pathology, growth, metastasis and disease outcomes. Nat Med 2011; 17: 1514–1520. 22019887 10.1038/nm.2454 PMC3553601

[18] Garrido‐Laguna I , Uson M , Rajeshkumar NV , *et al*. Tumor engraftment in nude mice and enrichment in stroma‐related gene pathways predict poor survival and resistance to gemcitabine in patients with pancreatic cancer. Clin Cancer Res 2011; 17: 5793–5800. 21742805 10.1158/1078-0432.CCR-11-0341 PMC3210576

[19] Moon HG , Oh K , Lee J , *et al*. Prognostic and functional importance of the engraftment‐associated genes in the patient‐derived xenograft models of triple‐negative breast cancers. Breast Cancer Res Treat 2015; 154: 13–22. 26438141 10.1007/s10549-015-3585-y

[20] Neale G , Su X , Morton CL , *et al*. Molecular characterization of the pediatric preclinical testing panel. Clin Cancer Res 2008; 14: 4572–4583. 18628472 10.1158/1078-0432.CCR-07-5090 PMC4209898

[21] Eirew P , Steif A , Khattra J , *et al*. Dynamics of genomic clones in breast cancer patient xenografts at single‐cell resolution. Nature 2015; 518: 422–426. 25470049 10.1038/nature13952 PMC4864027

[22] Monterrubio C , Pascual‐Pasto G , Cano F , *et al*. SN‐38‐loaded nanofiber matrices for local control of pediatric solid tumors after subtotal resection surgery. Biomaterials 2016; 79: 69–78. 26695118 10.1016/j.biomaterials.2015.11.055

[23] Nanni P , Landuzzi L , Manara MC , *et al*. Bone sarcoma patient‐derived xenografts are faithful and stable preclinical models for molecular and therapeutic investigations. Sci Rep 2019; 9: 12174. 31434953 10.1038/s41598-019-48634-y PMC6704066

[24] Bukchin A , Pascual‐Pasto G , Cuadrado‐Vilanova M , *et al*. Glucosylated nanomicelles target glucose‐avid pediatric patient‐derived sarcomas. J Control Release 2018; 276: 59–71. 29501533 10.1016/j.jconrel.2018.02.034

[25] Meehan TF , Conte N , Goldstein T , *et al*. PDX‐MI: minimal information for patient‐derived tumor xenograft models. Cancer Res 2017; 77: e62–e66. 29092942 10.1158/0008-5472.CAN-17-0582 PMC5738926

[26] Monterrubio C , Paco S , Vila‐Ubach M , *et al*. Combined microdialysis‐tumor homogenate method for the study of the steady state compartmental distribution of a hydrophobic anticancer drug in patient‐derived xenografts. Pharm Res 2015; 32: 2889–2900. 25773723 10.1007/s11095-015-1671-9

[27] Skaltsa K , Jover L , Carrasco JL . Estimation of the diagnostic threshold accounting for decision costs and sampling uncertainty. Biom J 2010; 52: 676–697. 20976697 10.1002/bimj.200900294

[28] Monterrubio C , Paco S , Olaciregui NG , *et al*. Targeted drug distribution in tumor extracellular fluid of GD2‐expressing neuroblastoma patient‐derived xenografts using SN‐38‐loaded nanoparticles conjugated to the monoclonal antibody 3F8. J Control Release 2017; 255: 108–119. 28412222 10.1016/j.jconrel.2017.04.016 PMC5564453

[29] Cosetti M , Wexler LH , Calleja E , *et al*. Irinotecan for pediatric solid tumors: the Memorial Sloan‐Kettering experience. J Pediatr Hematol Oncol 2002; 24: 101–105. 11990694 10.1097/00043426-200202000-00009

[30] Thompson J , Zamboni WC , Cheshire PJ , *et al*. Efficacy of systemic administration of irinotecan against neuroblastoma xenografts. Clin Cancer Res 1997; 3: 423–431. 9815701

[31] Ward ZJ , Yeh JM , Bhakta N , *et al*. Global childhood cancer survival estimates and priority‐setting: a simulation‐based analysis. Lancet Oncol 2019; 20: 972–983. 31129029 10.1016/S1470-2045(19)30273-6

[32] Winther JF , Kenborg L , Byrne J , *et al*. Childhood cancer survivor cohorts in Europe. Acta Oncol 2015; 54: 655–668. 25813473 10.3109/0284186X.2015.1008648

[33] Stewart E , Federico SM , Chen X , *et al*. Orthotopic patient‐derived xenografts of paediatric solid tumours. Nature 2017; 549: 96–100. 28854174 10.1038/nature23647 PMC5659286

[34] Morton CL , Houghton PJ . Establishment of human tumor xenografts in immunodeficient mice. Nat Protoc 2007; 2: 247–250. 17406581 10.1038/nprot.2007.25

[35] Nicolle D , Fabre M , Simon‐Coma M , *et al*. Patient‐derived mouse xenografts from pediatric liver cancer predict tumor recurrence and advise clinical management. Hepatology 2016; 64: 1121–1135. 27115099 10.1002/hep.28621

[36] Chen Y , Zhang R , Wang L , *et al*. Tumor characteristics associated with engraftment of patient‐derived non‐small cell lung cancer xenografts in immunocompromised mice. Cancer 2019; 125: 3738–3748. 31287557 10.1002/cncr.32366 PMC7294643

[37] Hernandez MC , Yang L , Leiting JL , *et al*. Successful secondary engraftment of pancreatic ductal adenocarcinoma and cholangiocarcinoma patient‐derived xenografts after previous failed primary engraftment. Transl Oncol 2019; 12: 69–75. 30273859 10.1016/j.tranon.2018.09.008 PMC6170258

[38] Brabetz S , Leary SES , Gröbner SN , *et al*. A biobank of patient‐derived pediatric brain tumor models. Nat Med 2018; 24: 1752–1761. 30349086 10.1038/s41591-018-0207-3

[39] Yu L , Baxter PA , Voicu H , *et al*. A clinically relevant orthotopic xenograft model of ependymoma that maintains the genomic signature of the primary tumor and preserves cancer stem cells in vivo. Neuro Oncol 2010; 12: 580–594. 20511191 10.1093/neuonc/nop056 PMC2940646

[40] Shu Q , Wong KK , Su JM , *et al*. Direct orthotopic transplantation of fresh surgical specimen preserves CD133+ tumor cells in clinically relevant mouse models of medulloblastoma and glioma. Stem Cells 2008; 26: 1414–1424. 18403755 10.1634/stemcells.2007-1009

[41] Okano M , Oshi M , Butash A , *et al*. Orthotopic implantation achieves better engraftment and faster growth than subcutaneous implantation in breast cancer patient‐derived xenografts. J Mammary Gland Biol Neoplasia 2020; 25: 27–36. 32109311 10.1007/s10911-020-09442-7 PMC7141774

[42] Siolas D , Hannon GJ . Patient‐derived tumor xenografts: transforming clinical samples into mouse models. Cancer Res 2013; 73: 5315–5319. 23733750 10.1158/0008-5472.CAN-13-1069 PMC3766500

[43] Sitt JC , Griffith JF , Lai FM , *et al*. Ultrasound‐guided synovial Tru‐cut biopsy: indications, technique, and outcome in 111 cases. Eur Radiol 2017; 27: 2002–2010. 27553941 10.1007/s00330-016-4545-6

[44] Braekeveldt N , Wigerup C , Gisselsson D , *et al*. Neuroblastoma patient‐derived orthotopic xenografts retain metastatic patterns and geno‐ and phenotypes of patient tumours. Int J Cancer 2015; 136: E252–E261. 25220031 10.1002/ijc.29217 PMC4299502

[45] Tentler JJ , Tan AC , Weekes CD , *et al*. Patient‐derived tumour xenografts as models for oncology drug development. Nat Rev Clin Oncol 2012; 9: 338–350. 22508028 10.1038/nrclinonc.2012.61 PMC3928688

[46] Ben‐David U , Ha G , Tseng YY , *et al*. Patient‐derived xenografts undergo mouse‐specific tumor evolution. Nat Genet 2017; 49: 1567–1575. 28991255 10.1038/ng.3967 PMC5659952

[47] Gerlinger M , Rowan AJ , Horswell S , *et al*. Intratumor heterogeneity and branched evolution revealed by multiregion sequencing. N Engl J Med 2012; 366: 883–892. 22397650 10.1056/NEJMoa1113205 PMC4878653

[48] Stewart CF , Zamboni WC , Crom WR , *et al*. Disposition of irinotecan and SN‐38 following oral and intravenous irinotecan dosing in mice. Cancer Chemother Pharmacol 1997; 40: 259–265. 9219511 10.1007/s002800050656

[49] Mora J , Cruz CO , Parareda A , *et al*. Treatment of relapsed/refractory pediatric sarcomas with gemcitabine and docetaxel. J Pediatr Hematol Oncol 2009; 31: 723–729. 19727011 10.1097/MPH.0b013e3181b2598c

[50] Wagner LM , McAllister N , Goldsby RE , *et al*. Temozolomide and intravenous irinotecan for treatment of advanced Ewing sarcoma. Pediatr Blood Cancer 2007; 48: 132–139. 16317751 10.1002/pbc.20697

[51] Strauss SJ , McTiernan A , Driver D , *et al*. Single center experience of a new intensive induction therapy for Ewing's family of tumors: feasibility, toxicity, and stem cell mobilization properties. J Clin Oncol 2003; 21: 2974–2981. 12885818 10.1200/JCO.2003.04.106

[52] Raciborska A , Bilska K , Drabko K , *et al*. Vincristine, irinotecan, and temozolomide in patients with relapsed and refractory Ewing sarcoma. Pediatr Blood Cancer 2013; 60: 1621–1625. 23776128 10.1002/pbc.24621

[53] Dharmarajan KV , Wexler LH , Wolden SL . Concurrent radiation with irinotecan and carboplatin in intermediate‐ and high‐risk rhabdomyosarcoma: a report on toxicity and efficacy from a prospective pilot phase II study. Pediatr Blood Cancer 2013; 60: 242–247. 22619050 10.1002/pbc.24205

[54] Meyers PA , Schwartz CL , Krailo MD , *et al*. Osteosarcoma: the addition of muramyl tripeptide to chemotherapy improves overall survival – a report from the Children's Oncology Group. J Clin Oncol 2008; 26: 633–638. 18235123 10.1200/JCO.2008.14.0095

[55] Ferrari S , Smeland S , Mercuri M , *et al*. Neoadjuvant chemotherapy with high‐dose ifosfamide, high‐dose methotrexate, cisplatin, and doxorubicin for patients with localized osteosarcoma of the extremity: a joint study by the Italian and Scandinavian Sarcoma Groups. J Clin Oncol 2005; 23: 8845–8852. 16246977 10.1200/JCO.2004.00.5785

