# Supplementary material for: Prognostic value of patient‐derived xenograft engraftment in pediatric sarcomas
Source: J Pathol Clin Res. 2021 Apr 9;7(4):338–49. doi: 10.1002/cjp2.210 (PMC8185364; doi:10.1002/cjp2.210)
Supplement: Supplementary file 1 — Figure S1. Staining of human cells in six representative cases of xenografts at early and late passages Figure S2. Comparative histology of two PDX from the same rhabdomyosarcoma patient at relapse and necropsy Figure S3. Analysis of cell proliferation in human tumor biopsies and their corresponding PDXs Figure S4. Whole‐genome visualization of the chromosomal profiles of patients with Ewing sarcoma and rhabdomyosarcoma and their matched PDX models Table S1. Clinical data of patients with Ewing sarcoma, osteosarcoma, and rhabdomyosarcoma Table S2. Data from PDX established from patients with Ewing sarcoma, osteosarcoma, and rhabdomyosarcoma Table S3. Clinical information from patients with Ewing sarcoma (N = 31; median age: 12.3 years; range: 0.52–17.9 years) Table S4. Clinical information from patients with osteosarcoma (N = 10; median age: 11.1 years; range: 5.67–14.7 years) Table S5. Clinical information from patients with rhabdomyosarcoma (N = 10; median age: 9.7 years; range: 4.03–15.4 years) Table S6. Engraftment outcomes of patient samples Table S7. Association of technical factors with engraftment Table S8. Association of previous biopsy engraftment with the engraftment of the following biopsy obtained from the same patient Table S9. Association of patient factors with engraftment in patients with Ewing sarcoma Table S10. Clinical data from PDX models included in the efficacy study of irinotecan Table S11. PDX response to treatment with a single cycle of irinotecan Table S12. Comparative response to irinotecan‐based treatments of rhabdomyosarcoma patients and their corresponding PDX Table S13. Comparative response to irinotecan treatment and median survival of PDXs at F ≤ 2 or F ≥ 6 passages [file CJP2-7-338-s001.pdf]

Prognostic value of patient-derived xenograft engraftment in pediatric sarcomas
H Castillo-Ecija *et al*. *J Pathol Clin Res* DOI: 10.1002/cjp2.210

Supplementary Material

Reference numbers refer to the list in the main paper

Figure S1. Staining of human cells in six representative cases of xenografts at early and late passages

Figure S2. Comparative histology of two PDX derived from the same rhabdomyosarcoma patient at relapse and necropsy

Figure S3. Analysis of cell proliferation in human tumor biopsies and their corresponding PDXs

Figure S4. Whole genome visualization of the chromosomal profiles of patients with Ewing sarcoma and rhabdomyosarcoma and their matched PDX models

Table S1. Clinical data of patients with Ewing sarcoma, osteosarcoma and rhabdomyosarcoma

Table S2. Data from patient derived-xenografts (PDX) established from patients with Ewing sarcoma, osteosarcoma and rhabdomyosarcoma

Table S3. Clinical information from patients with Ewing sarcoma (N = 31; median age 12.3 years; range 0.52 – 17.9 years)

Table S4. Clinical information from patients with osteosarcoma (N = 10; median age 11.1 years; range 5.67 – 14.7 years)

Table S5. Clinical information from patients with rhabdomyosarcoma (N = 10; median age 9.7 years; range 4.03 – 15.4 years)

Table S6. Engraftment outcomes of patient samples

Table S7. Association of technical factors with engraftment

Table S8. Association of previous biopsy engraftment with the engraftment of the following biopsy obtained from the same patient

Table S9. Association of patient factors with engraftment in patients with Ewing sarcoma

Table S10. Clinical data from PDX models included in the efficacy study of irinotecan

Table S11. PDX response to treatment with a single cycle of irinotecan

Table S12. Comparative response to irinotecan-based treatments of rhabdomyosarcoma patients and their corresponding PDX

Table S13. Comparative response to irinotecan treatment and median survival of PDXs at $F \leq 2$ or $F \geq 6$ passages

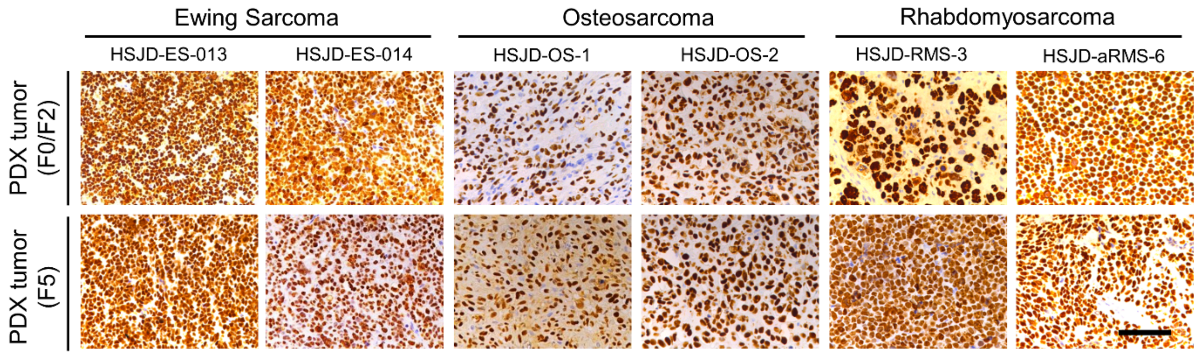

Figure S1. Staining of human cells (human nuclei, in brown) in six representative cases of xenografts at early (*F0*/*F2*) and late passages (*F5*). These representative samples were selected among six Ewing sarcoma, three osteosarcoma and three rhabdomyosarcoma with complete histopathology studies. All images were obtained using a microscope with a 20x objective. Scale bar represents 50 µm.

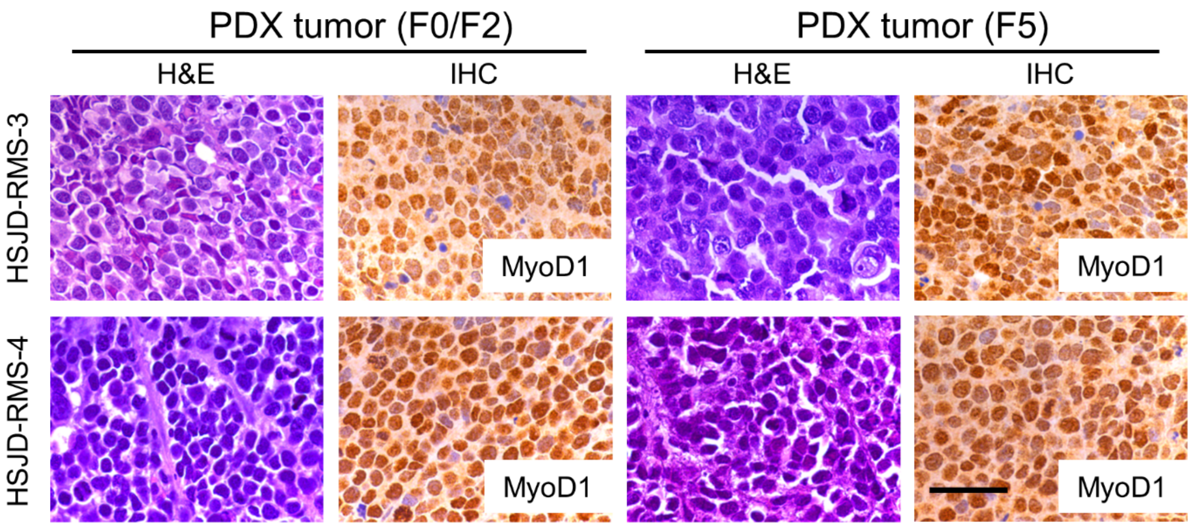

Figure S2. Comparative histology (H&E and MyoD1 staining) of two PDX derived from the same rhabdomyosarcoma patient at relapse (HSJD-RMS-3) and necropsy (HSJD-RMS-4). MyoD1 (nuclear) is stained in brown. All images were obtained using a microscope with a 40x objective. Scale bar represents 25 μm.

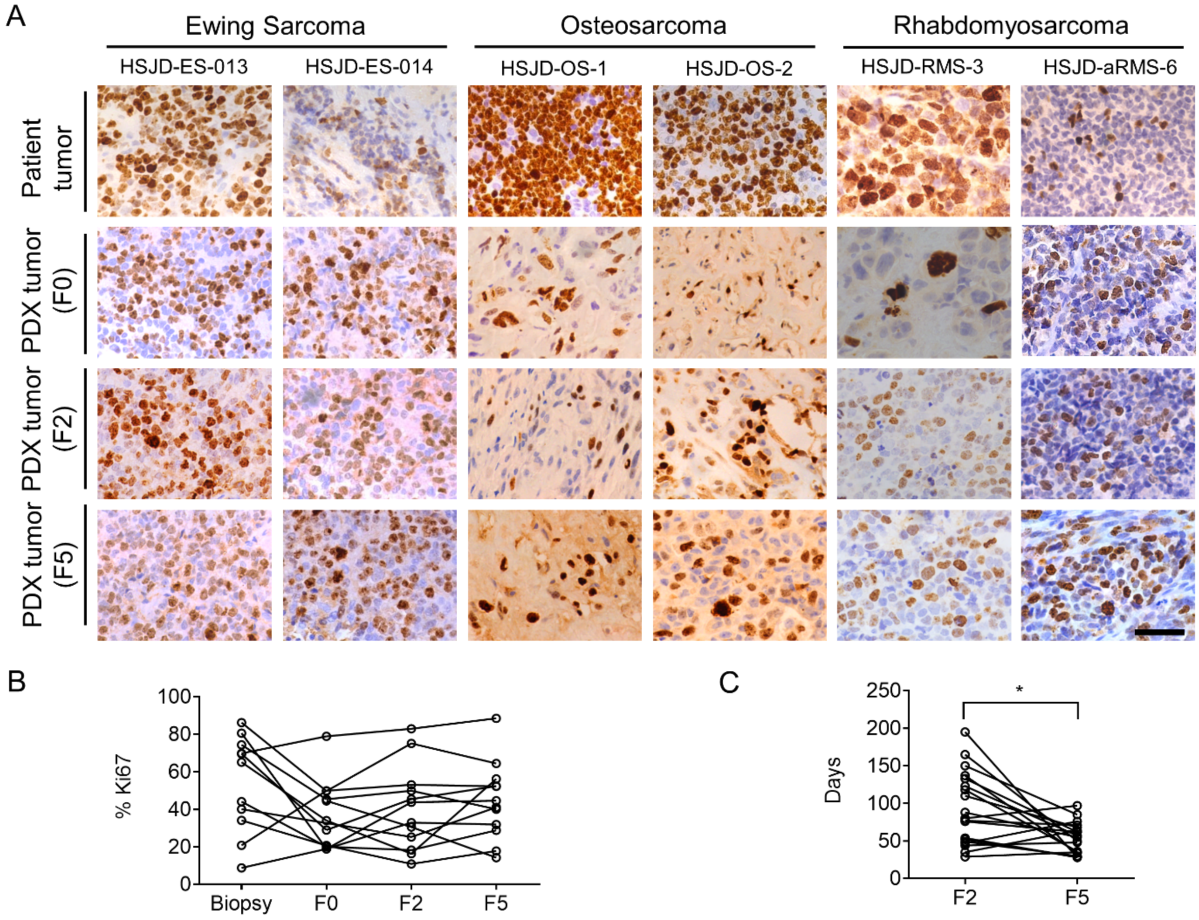

Figure S3. Analysis of cell proliferation in human tumor biopsies and their corresponding PDXs. (A) Ki67 histology staining in patient tumor and three PDX generations (*F0*, *F2* and *F5*) in six representative cases. All images were obtained using a microscope with a 40x objective. Scale bar represents 25 µm. (B) Quantification of Ki67 positive cells (%). These samples were selected among six Ewing sarcoma, three osteosarcoma and three rhabdomyosarcoma with complete histopathology studies. Each point represents the mean of ten slides per tumor and generation. (C) Time in days of tumor growth at *F2* and *F5* to achieve 1500 mm^3. *P = 0.0042, paired t test, *F2* vs *F5*.

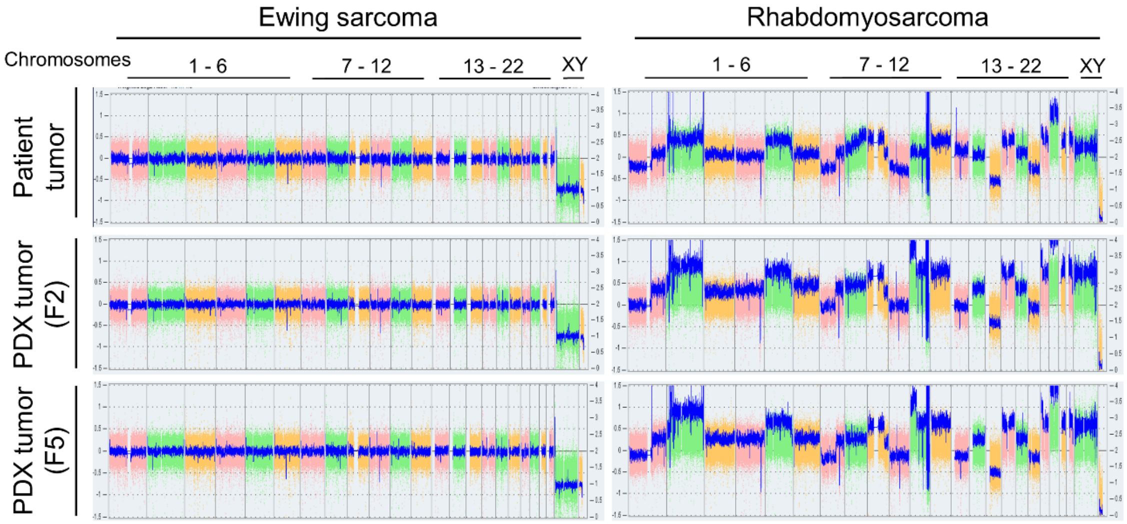

Figure S4. Whole genome visualization of the chromosomal profiles of patients with Ewing sarcoma (model named HSJD-ES-012) and rhabdomyosarcoma (HSJD-RMS-4) and their matched PDX models at generations *F2* and *F5*. The log2 copy number track and the smooth signal indicate the segmental and copy number aberrations observed in the samples. Each column represents a different chromosome.

Table S1. Clinical data of patients with Ewing sarcoma, osteosarcoma and rhabdomyosarcoma

Data type	Description	Entry (example)
Patient clinical information	Patient ID	ES-009
	Gender	Male
	Age	12.5 years old
	Diagnosis	Ewing sarcoma
	Relapse, dead or last follow up date	20/10/2010
	Previous treatment	GEIS21
	Treatment response	Complete (RECIST protocol)
Tumor information	Tumor ID	B20-01234
	Biopsy date	01/01/2020
	Primary tumor tissue of origin	Chest wall and ribs
	Timing of surgery	Relapse
	Tissue histology	H&E and IHC panel
	Molecular tissue analysis	Fusion gene *EWSR1-FLI1*
	Sample collection method	Tru-Cut needle

Table S2. Data from patient derived-xenografts (PDX) established from patients with Ewing sarcoma, osteosarcoma and rhabdomyosarcoma

Data type	Description	Entry (example)
Xenograft establishment	PDX ID	HSJD-ES-009
	Mouse strain (source)	NOD-SCID (Envigo)
	Sample conservation	Freshly excised
	Inoculation type and site	Subcutaneous; both flanks
	Use of Matrigel	No
	Engraftment time	4.2 months
Tumor analysis	Tumor characterization technology	Histology and IHC panel
	Anti-human nuclei staining	Positive
Model study	Passage	*F0*
	Treatment 1	Irinotecan 10 mg/kg/day; 5-day-on-2-off
	Response to treatment 1	CR

Table S3. Clinical information from patients with Ewing sarcoma (N = 31; median age 12.3 years; range 0.52 – 17.9 years)

**Factor**	**No. of Patients**	**%**
Gender		
Female	8	25.8
Male	23	74.2
Fusion gene type		
EWSR1-ERG	2	6.5
EWSR1-FLI1	29	93.5
Primary tumor		
Chest wall and ribs	6	19.4
Limbs	12	38.7
Pelvic bones	6	19.4
Vertebral spine	7	22.6
Metastasis at diagnosis		
No	23	74.2
Yes	8	25.8
Risk stratification of newly diagnosed patients[1]		
Standard	7	38.9
High	11	61.1

[1]Of the 31 patients included in this study, 18 were included at disease diagnosis. For such patients, we applied the risk stratification protocol published in reference [5].

Table S4. Clinical information from patients with osteosarcoma (N = 10; median age 11.1 years; range 5.67 – 14.7 years)

Factor	No. of Samples	%
Gender		
Female	3	30
Male	7	70
Primary tumor		
Limbs	10	100
Metastasis at diagnosis		
No	8	80
Yes	2	20

Table S5. Clinical information from patients with rhabdomyosarcoma (N = 10; median age 9.7 years; range 4.03 – 15.4 years)

**Factor**	**No. of Samples**	**%**
Gender		
Female	3	30
Male	7	70
Tumor type		
Alveolar (aRMS)	5	50
Embryonal (eRMS)	5	50
Fusion gene type		
Absent	5	50
PAX3-FKHR	4	40
PAX7-FKHR	1	10
Primary tumor		
Chest wall and ribs	1	10
Head and neck	3	30
Limbs	3	30
Pelvic bones	1	10
Urogenital region	2	20
Metastasis at diagnosis		
No	6	60
Yes	4	40

Table S6. Engraftment outcomes of patient samples

Patient	Disease	Biopsies attempted	Biopsies engrafted	PDX ID
1	Ewing sarcoma	1	1	HSJD-ES-004
2	Ewing sarcoma	1	1	HSJD-ES-003
3	Osteosarcoma	1	0	None
4	Ewing sarcoma	1	0	None
5	Ewing sarcoma	1	0	None
6	Rhabdomyosarcoma	2	1	HSJD-aRMS-5
7	Ewing sarcoma	1	0	None
8	Rhabdomyosarcoma	1	1	HSJD-aRMS-6
9	Ewing sarcoma	2	0	None
10	Rhabdomyosarcoma	3	2	HSJD-aRMS-1, HSJD-aRMS-2
11	Ewing sarcoma	1	0	None
12	Ewing sarcoma	2	0	None
13	Ewing sarcoma	2	2	HSJD-ES-005, HSJD-ES-016
14	Rhabdomyosarcoma	2	1	HSJD-aRMS-7
15	Ewing sarcoma	1	0	None
16	Ewing sarcoma	2	2	HSJD-ES-001, HSJD-ES-007
17	Ewing sarcoma	3	0	None
18	Ewing sarcoma	1	0	None

19	Rhabdomyosarcoma	1	0	None
20	Osteosarcoma	2	1	HSJD-OS-4
21	Ewing sarcoma	1	0	None
22	Ewing sarcoma	1	0	None
23	Ewing sarcoma	1	0	None
24	Rhabdomyosarcoma	1	1	HSJD-aRMS-8
25	Ewing sarcoma	2	2	HSJD-ES-002, HSJD-ES-006
26	Ewing sarcoma	1	0	None
27	Ewing sarcoma	1	0	None
28	Ewing sarcoma	2	2	HSJD-ES-009, HSJD-ES-018
29	Osteosarcoma	2	2	HSJD-OS-1, HSJD-OS-5
30	Rhabdomyosarcoma	2	2	HSJD-RMS-3, HSJD-RMS-4
31	Ewing sarcoma	1	0	None
32	Ewing sarcoma	1	1	HSJD-ES-015
33	Ewing sarcoma	1	0	None
34	Ewing sarcoma	2	2	HSJD-ES-008, HSJD-ES-012
35	Osteosarcoma	1	0	None
36	Ewing sarcoma	1	1	None
37	Ewing sarcoma	2	2	HSJD-ES-013, HSJD-ES-017
38	Ewing sarcoma	1	1	HSJD-ES-014
39	Rhabdomyosarcoma	1	0	None

40	Osteosarcoma	1	0	None
41	Ewing sarcoma	1	0	None
42	Osteosarcoma	1	1	HSJD-OS-2
43	Ewing sarcoma	1	0	None
44	Osteosarcoma	1	0	None
45	Osteosarcoma	1	1	HSJD-OS-3
46	Ewing sarcoma	1	0	None
47	Osteosarcoma	1	0	None
48	Osteosarcoma	1	0	None
49	Rhabdomyosarcoma	1	0	None
50	Rhabdomyosarcoma	1	0	None
51	Ewing sarcoma	1	0	None

Table S7. Association of technical factors with engraftment

Factor	No. of Samples	% Engrafted	No. of Engrafted	OR (95% CI)	*P*
Sample conservation					
Freshly excised	56	44.6	25		
Cryopreserved	12	41.7	5	1.28 (0.3 to 6.2)	0.76
Use of Matrigel					
No	41	43.9	18		
Yes	27	44.4	12	1.13 (0.3 to 3.9)	0.84
Biopsy method					
Tru-cut needle	32	31.2	10		
Surgery	35	57.1	20	4.05 (1-17.3)	0.059

Table S8. Association of previous biopsy engraftment with the engraftment of the following biopsy obtained from the same patient

Factor	No. of Samples	% Engrafted	No. of Engrafted	OR (95% CI)	*P*
PDX already established from previous biopsy					
Yes	11	82	9		
No	4	25	1	13.5 (1.13 to 18)	0.0769

Table S9. Association of patient factors with engraftment in patients with Ewing sarcoma

Factor	No. of Samples	% Engrafted	No. of Engrafted	Odds ratio (95% CI)	*P*
Age, years					
< 12	18	22.2	4		
≥ 12	23	56.5	13	4.33 (0.00 – 748.21)	0.70
Timing of surgery					
Diagnosis	18	22.2	4		
Relapse	23	56.5	13	4.76 (4.74 to 4.78)	<0.0001
Biopsy origin					
Limbs	11	36.4	4		
Head and neck	7	71.4	5	$2.44 \cdot 10^{9}$ (534.56 – 1.11·1016)[a]	
Chest wall and ribs	5	40.0	2	2.86 ($9.83 \cdot 10^{-5}$ – $8.29 \cdot 10^{4}$)[a]	
Lung or pleura	9	55.6	5	$6.80 \cdot 10^{8}$ (2081.25 – $2.22 \cdot 10^{14}$)[a]	
Pelvic bones	3	33.3	1	1.20 ($4.65 \cdot 10^{-6}$ – $3.10 \cdot 10^{5}$)[a]	0.52
Muscle	0	0	0	*	
Teste	0	0	0	*	
Vertebral spine	6	0	0	*	
Metastasis at diagnosis					
No	29	31.0	9		
Yes	12	66.7	8	$6.62 \cdot 10^{9}$ ($6.59 \cdot 10^{4}$ – $6.65 \cdot 10^{14}$)	0.00012

*Excluded from statistical analysis of biopsy origin due to lack of an event in No. of engrafted or in No. of nonengrafted samples.
[a]Compared to limbs.

Table S10. Clinical data from PDX models included in the efficacy study of irinotecan

PDX model ID	Disease stage	Age (years) (g)[a]	Primary	Metastasis at diagnosis	Previous treatment	Biopsy location	Fusion gene type
HSJD-ES-001	Relapse	17.8 (M)	Scapula	Lung, skull, ribs, sternum, bone marrow	G/D[b], I/T[c], RT[d], HIFU[e]	Scapula	*EWSR1-FLI1* fusion (type II)
HSJD-ES-002	Diagnosis	12.2 (M)	Fibula	None	None	Fibula	*EWSR1-FLI1* fusion (ex10-ex5)
HSJD-ES-004	Relapse	18 (M)	Mediastinum	None	SEHOP 2001[f]	Mediastinum	*EWSR1-FLI1* fusion (type I)
HSJD-ES-006	Relapse	13.5 (M)	Fibula	Pleura, lung	GEIS21 (standard risk)[g]	Pleura	*EWSR1-FLI1* fusion (ex10-ex5)
HSJD-ES-008	Diagnosis	13 (M)	Humerus	Lung, pleura, lymph node, bone, bone marrow	None	Humerus	*EWSR1-FLI1* fusion (type I)
HSJD-ES-009	Relapse	10.7 (M)	Chest	None	GEIS 21 (standard risk)[g]	Skull	*EWSR1-FLI1* fusion (type II)

HSJD-ES-011	Relapse	13.9 (F)	Ribs	None	SEHOP 2001[f], VIT[h]	Pleura	*EWSR1-FLI1* fusion (type I)
HSJD-ES-012	Relapse	14.7 (M)	Humerus	Skull	GEIS21 (high risk)[i]; I/T[c]; G/D[b]	Skull	*EWSR1-FLI1* fusion (type I)
HSJD-ES-013	Relapse	18.6 (M)	Pleura	None	GEIS21 (high risk)[i]	Trapezius	*EWSR1-FLI1* fusion (type I)
HSJD-ES-015	Relapse	18.3 (M)	Tibia	None	GEIS21 (standard risk)[g]	Lung	*EWSR1-FLI1* fusion (type I)
HSJD-ES-017	Diagnosis	17.9 (M)	Pleura	None	None	Pleura	*EWSR1-FLI1* fusion (type I)
HSJD-aRMS-1	Relapse	12.3 (M)	Ribs	None	MSKCC[j]	Ribs	*PAX3-FKHR* fusion
HSJD-aRMS-2	Relapse	14 (M)	Ribs	None	MSKCC[j]	Pleura	*PAX3-FKHR* fusion

HSJD-RMS-3	Relapse	8.3 (F)	Maxillary bone	Parotid gland, maxillary sinus, orbital bone	MSKCC[j]	Lymph node	Absent
HSJD-aRMS-7	Diagnosis	11.1 (M)	Thigh	None	None	Thigh	*PAX7-FKHR* fusion
HSJD-RMS-9	Diagnosis	1.4 (M)	Retroperitoneum	None	None	Retroperitoneum	Absent
HSJD-RMS-11	Diagnosis	12.3 (M)	Perianal	Mediastinum, lung, retroperitoneum, lymph node	None	Perianal	Absent
HSJD-OS-1	Relapse	15.5 (M)	Tibia	None	SEHOP-SO-2010[k]	Lung	-
HSJD-OS-4	Relapse	15.4 (M)	Femur	None	SEHOP-SO-2001[l]	Thigh	-

[a]g: gender. M: male; F: female.
[b]G/D: clinical protocol including gemcitabine and docetaxel as in reference [49].
[c]I/T: clinical protocol including irinotecan and temozolomide as in reference [50].
[d]RT: radiation therapy.
[e]HIFU: high intensity focused ultrasound.
[f]SEHOP 2001: clinical protocol including six cycles of VIDE chemotherapy (day 1 vincristine followed by day 1 to 3 with doxorubicin, ifosfamide and etoposide) as in reference [51].

[g]GEIS21 (standard risk): clinical protocol including five cycles of mP6 chemotherapy (cycles 1, 2 and 4 with cyclophosphamide, doxorubicin and vincristine; and cycles 3 and 5 with ifosfamide and etoposide); surgery; radiation therapy as in reference [5].
[h]VIT: clinical protocol including vincristine, irinotecan and temozolomide as in reference [52].
[i]GEIS21 (high risk): clinical protocol including gemcitabine/docetaxel (G/D) window phase, followed by five cycles of mP6 chemotherapy; surgery; radiation therapy; followed by G/D maintenance therapy as in reference [5].
[j]MSKCC: clinical protocol at Memorial Sloan-Kettering Cancer Center, including two cycles of carboplatin and irinotecan window phase, followed by three cycles of vincristine, doxorubicin and cyclophosphamide (VAdriaC) induction therapy; radiation therapy and surgery as in reference [53].
[k]SEHOP-SO-2010: clinical protocol including two cycles of high-dose ifosfamide, methotrexate, cisplatin and doxorubicin followed by surgery. After surgery, liposomal-muramyl tripeptide phosphatidyl-ethanolamine (MEPACT) followed by two cycles of doxorubicin and three cycles of high-dose ifosfamide, methotrexate and cisplatin as in reference [54].
[l]SEHOP-SO-2001: clinical protocol including two cycles of high-dose ifosfamide, methotrexate, cisplatin and doxorubicin followed by surgery. Post-surgery treatment consisted in two cycles of doxorubicin and three cycles of high-dose ifosfamide, methotrexate and cisplatin as in reference [55].

Table S11. PDX response to treatment with a single cycle of irinotecan

PDX model	Generation	Treatment	N (tumors)	CR (%)	PR (%)	SD (%)	PD (%)
HSJD-ES-001	*F6*	Control	5	0	0	0	100
		Irinotecan	6	100	0	0	0
HSJD-ES-002	*F4*	Control	8	0	0	0	100
		Irinotecan	6	100	0	0	0
HSJD-ES-004	*F4*	Control	6	0	0	0	100
		Irinotecan	7	100	0	0	0
HSJD-ES-006	*F3*	Control	6	0	0	0	100
		Irinotecan	6	100	0	0	0
HSJD-ES-008	*F3*	Control	6	0	0	0	100
		Irinotecan	6	100	0	0	0
HSJD-ES-011	*F6*	Control	3	0	0	0	100
		Irinotecan	7	0	0	100	0
HSJD-ES-012	*F4*	Control	4	0	0	0	100
		Irinotecan	5	0	0	100	0
HSJD-ES-013	*F4*	Control	5	0	0	0	100
		Irinotecan	5	100	0	0	0
HSJD-ES-015	*F4*	Control	7	0	0	0	100
		Irinotecan	7	0	28	72	0
HSJD-aRMS-1	*F10*	Control	7	0	0	0	100
		Irinotecan	7	14	72	14	0
HSJD-RMS-3	*F11*	Control	6	0	0	0	100
		Irinotecan	7	100	0	0	0
HSJD-aRMS-7	*F5*	Control	4	0	0	0	100
		Irinotecan	3	0	100	0	0
HSJD-RMS-9[a]	*F3*	Control	7	0	0	0	100
		Irinotecan	7	100	0	0	0
HSJD-RMS-11[a]	*F2*	Control	5	0	0	0	100
		Irinotecan	5	100	0	0	0
HSJD-OS-1	*F4*	Control	5	0	0	0	100
		Irinotecan	9	11	33	56	0
HSJD-OS-4	*F1*	Control	4	0	0	0	100
		Irinotecan	3	0	0	100	0

[a]Model obtained from patient not included in the PDX engraftment study cohort

Table S12. Comparative response to irinotecan-based treatments of rhabdomyosarcoma patients and their corresponding PDX

PDX model ID	Patient Treatment	Clinical response	PDX response to irinotecan
HSJD-aRMS-7	Irinotecan plus carboplatin	PR	PR
HSJD-RMS-9	Irinotecan plus vincristine	SD	CR
HSJD-aRMS-10	Irinotecan plus carboplatin	PR	PR
HSJD-RMS-11	Irinotecan plus carboplatin	CR	CR

Table S13. Comparative response to irinotecan treatment and median survival of PDXs at *F*≤ *2* or *F* ≥ *6* passages

PDX model	*F(x)*	Treatment	N (tumors)	CR (%)	PR (%)	SD (%)	PD (%)	Median survival (days)	*P*[a]
HSJD-ES-009	*F2*	Control	6	0	0	0	100	16	
		Irinotecan	7	14	14	72	0	>30[b]	0.0008
	F6	Control	10	0	0	0	100	21	
		Irinotecan	9	11	22	56	11	44	<0. 0001
HSJD-ES-017	*F1*	Control	8	0	0	0	100	51.5	
		Irinotecan	8	50	13	37	0	84	0.00014
	F8	Control	5	0	0	0	100	56	
		Irinotecan	7	0	72	28	0	88	0.0016
HSJD-aRMS-2	*F2*	Control	3	0	0	0	100	48	
		Irinotecan	3	100	0	0	0	86	0.0246
	F10	Control	6	0	0	0	100	31	
		Irinotecan	6	33	67	0	0	88.5	0.0005

[a]*P* value, compared to control.
[b]This study was ceased at day 30.
